# Does competitive position matter: Investigating the impact of information risk on COE and corporate investment

**DOI:** 10.1371/journal.pone.0322527

**Published:** 2025-05-27

**Authors:** Sana Saleem, Muhammad Usman, Muhammad Naveed Akhtar

**Affiliations:** 1 Department of Business Education, The University of Chenab, Gujrat, Pakistan; 2 Department of Management Sciences, University of Gujrat, Gujrat, Pakistan; 3 Lahore Business School, University of Lahore, Lahore, Pakistan; Bucharest University of Economic Studies: Academia de Studii Economice din Bucuresti, ROMANIA

## Abstract

**Purpose:**

The purpose of this study is to find out how firm’s competitive position plays the moderating role between the relation of information risk and COE. The study considers the effect of two different types of information risk, i.e., lack of information quality and transparent information.

**Methodology:**

The data of the study is collected from all the non-financial firms listed on PSX from 2007 to 2022. Two-step system GMM dynamic panel estimators are applied to test the dynamic nature of the proposed model.

**Findings/Results:**

The findings show that firms having better competitive position signal their strength through improved information disclosure in order to gain the confidence of shareholders. This competitive environment poses a governance effect by imposing discipline on manager’s behavior, reducing information asymmetry and improving the quality of information disclosure, resulting in reduction of the COE. Further, a more competitive environment improves the readability of the annual report and reduces information asymmetry. In addition, by reducing financing frictions, this research provides new and unique insights pertaining to the importance of competitive position in the sensitivity of investment to information risk.

**Originality:**

This research extends to the corpus of literature by investigating the unexplored strategic determinants, such as a firm’s competitive position, to mitigate the impact of two distinct types of information risk: lower quality and reduced transparency. Additionally, it explores how these risks influence both the cost of equity and corporate investment.

## 1. Introduction

Does information risk matter? This seemingly fundamental question has sparked a heated debate. Traditional asset pricing models reveal that investors are only compensated for owning market risks. So, information risk should not be valued because it is expected that all-important information is always reflected in the stock prices. Nevertheless, this static perspective of market efficiency is incompatible with the dynamics of new information arrival [1]. In reality, when it comes to elimination of information risk as a factor in determining the required rate of return, Easley and O’Hara [2] argument convinces “*if the information is vital for the market, why shouldn’t it be necessary for the companies that participate in it?”*

Easley and O’Har*a’*s [3], remark is consistent with those made by legislators and standard-setters. When there is less uncertainty, there is less risk, and hence a lesser premium is demanded. Thus, the eventual effect of better transparency in the context of financial information is a lower cost of capital. Therefore, information is critical from the perspective of regulators and standard setters when it comes to determine required returns. However, similar to the theoretical literature, empirical research attempting to connect the information risk with cost of equity [COE] has not been able to provide convincing evidence. Due to the scantiness of agreement in the literature, the impact of information risk on COE remains an empirical subject. This research contributes to the discussion by investigating two forms of information risk.

The first form is information imprecision; specifically, the quality of information about the predicted cash flows [4]. In today’s digital era, despite the abundance of information accessible through various channels such as the internet, social media, and traditional print media, the sheer quantity does not guarantee its quality. This reality is underscored by numerous business scandals, exemplified by cases like the Islamic Investment Bank, Taj Firm, Mehran Bank, KASB Bank, and Axact. Even regulatory bodies, financial analysts, and investors were unable to foresee these financial irregularities. The inadequate explanatory power of financial data contributes to a lack of trust in the stock market [5]. Consequently, stakeholders’ confidence in publicly available financial information diminishes, leading them to demand higher returns and driving up the cost of equity [COE].

In light of [6–8] which highlighted the second form of information risk which arises due to less transparency; the extent to which financial statements disclose fundamentals in a way that is easily understood by individuals. With respect to information asymmetry, companies with complex and harder-to-read annual reports are more exposed to the risk of adverse selection glitches [9,10]. Previous studies have shown that the linguistic characteristics of annual reports have an impact on the long-term economic value of companies.

Considering above-mentioned forms of information risk and their effect on COE, this study highlights an important determinant of information disclosure, the firm’s competitive position and posits a question: *Does firm’s competitive position matter*? Literature has focused on various corporate governance disclosure mechanisms, as well as firm-specific characteristics. These studies focus on the quantity of disclosure but the present study considers the strategic dimension of disclosure, i.e., firm’s competitive position which focuses on what is disclosed and how much is disclosed.

According to the signaling hypothesis, firms in a favorable competitive position may seek to enhance their disclosures to communicate their efficiency. Investors interpret this disclosure as an indication of lower risk, resulting in a decline in COE. While partisans of agency theory argue that more disclosures would minimize information asymmetry [6,11–13]. This enables firms to raise capital at a minimal cost [14,15]. Therefore, competitive position improves not only the efficiency of information disclosure but also investors’ capacity to examine and comprehend it. This escalates information transparency while lowering the COE.

Further, the role of firm’s competitive position in the relationship between information risk and corporate investment decisions is also investigated in this study. It is expected that information risk has more substantial influence on Investment-Q sensitivity in highly competitive firms than in less competitive firms. Companies willing to compete are more liberal and transparent in their disclosures to assure their access to critical financial resources. This enhances investors trust, lowers agency costs and helps enterprises obtain external finance at a reduced price.

In the context of Pakistan, exploring the role of firm’s competitive position in information risk management is meaningful. Despite the improvements in institutional and regulatory norms, Pakistan’s financial markets are still afflicted by poor information structures. Destitute financial information quality, fiasco to report company-specific details and low transparency have major repercussions for the COE [16]. Due to this uncertain and weak information environment, investors demand high premium which increases the COE [16]. High COE prevents the firm from capturing investment opportunities, resulting inefficient investment decisions [17,18]. The majority of enterprises operating in Pakistan have a structure of concentrated ownership and unstructured BODs, which is the cause of these malfunction conditions [19–22]. Firms’ competitive position acts as a robust governance mechanism in this situation which improves the quality of disclosure of the company.

Therefore, this study adds value to the existing body of knowledge by supplementing the following studies. According to [23,24], accrual quality is not a priced factor and does not affect COE. McInnis [25] examined that earning smoothing does not reduce the COE. [26] investigated the role of conditional conservatism in lowering COE. According to [27,28], segment disclosure reduces COE, but this relationship is not more pronounced when there is strong market competition. Similarly, (96) studies the relationship between information risk and COE by considering the role of stock price crash risk while this research enhances to the burgeoning line of literature on competitive position and information disclosure by applying the [29] “approach to Pakistani stock market”. Unlike [29], this study investigates the moderating effect of firm’s competitive position on the relationship between information risk and COE by examining the two unique forms of information risk, i.e., less reporting quality and transparency in minimizing information asymmetry. In contrast to [27] and other earlier studies which used product market competition as a measure of competition, this study employs the broader concept of firm market status as a measure of competition. Several studies have shown competitive position as a significant driver of disclosures [30]. This study scrutinizes the moderating effect of competitive position by applying two different proxies of competitive position to perform the robust analysis. Second, the importance of a firm’s competitive position regarding information risk and investment sensitivity has also been investigated in this study. Third, by integrating signaling hypothesis and agency theory, the findings of this study advance theoretical understanding of how competitive position lessens information asymmetry, decreases COE and optimizes investment efficiency, specifically in developing country like Pakistan. Practically, firms can augment transparency and governance to cut down financing cost and attract capital. This is relevant especially in case of Pakistan’s volatile market, where strong industry positions can signify reliability to investors. Policymakers can harness these insights to bolster the robustness of financial reporting practices and governance practices, stimulating market growth. The study also underscores the need to address systemic risks to enhance investor confidence and ensure market efficiency.

## 2. Literature review and hypothesis development

The agency theory helps to understand how information risk and COE are related. The relationship between the managers and the shareholders is at the heart of agency theory [31]. Typically, the principal [owners] delegate managing and controlling authority to the [agent] management [32,33]. However, management is driven by their own short-term benefits and opportunities [34]. Managers/insiders may mislead external investors by providing self-discretionary disclosures [10,35,36]. Especially in underdeveloped nations like Pakistan, where inadequate regulations, ineffective law enforcement, and entrenched political influences make it difficult to prohibit, identify, and castigate opportunistic disclosures, this situation can be even more problematic [37–39]. As a result of this weak and uncertain information environment, there is an agency conflict and a high COE.

Traditional corporate governance mechanisms, on the other hand, have a long history of failing to shelter the benefits of minority shareholders [40,41]. Despite this, many firms with strong competitive position in the market perform efficiently and effectively [42–44]. Firm’s competitive position has a significant impact on corporate decisions. Therefore, this study looks into the effect of firms’ competitive position on the relationship between information risk and COE.

Several studies have identified the importance of competition in reducing information risk. Market competition reduces information asymmetry and limits managers’ opportunistic behavior in financial disclosure [45,46]. [45,47] contended that competition defends shareholders from embezzlement as competitive markets push managers to surpass competitors or face job dismissal and eventual insolvency [48–50]. [51] goes on to say that companies in the similar sector are subject to common productivity. By revealing performance benchmark across firms, competition increases transparency, which lessen information asymmetry and reduces the COE for well performing firms. Conforming to [51–53], competition reduces inefficient resource allocation in firms having weak governance structure, by curtailing managerial slacks. Appropriately, market competition is positively [negatively] associated with corporate disclosure.

Whereas, according to [54,55] Earning expectations are less numerous, horizons are shorter, transparency scores are lower, and information environments are opaque for companies in highly concentrated sectors. Competition is likely to lower predictable profits while increasing the risk of default [56–58]. Empirical manifest reveals that competition’s risk-increasing effect has important consequences for business actions such as hedging [59,60], financing [61], and payout policies [62].

“Contrarily, according to Resource dependent theory [RDT], firms, having greater reliance on external financing, face greater scrutiny from investors, and a strong competitive position can reduce this dependence by generating sufficient internal funds, which lowers COE. Similarly, firms that maintain legitimacy by adhering to societal and regularity expectations often exhibit higher transparency, reducing information asymmetry and perceived risk. This leads to a lower COE as investors associate legitimacy with reduced financial and operational risks. A legitimate firm is less likely to face financial penalties, regulatory interventions or reputational damage, reducing the risk premium demanded by equity investors.

**Fig 1:**
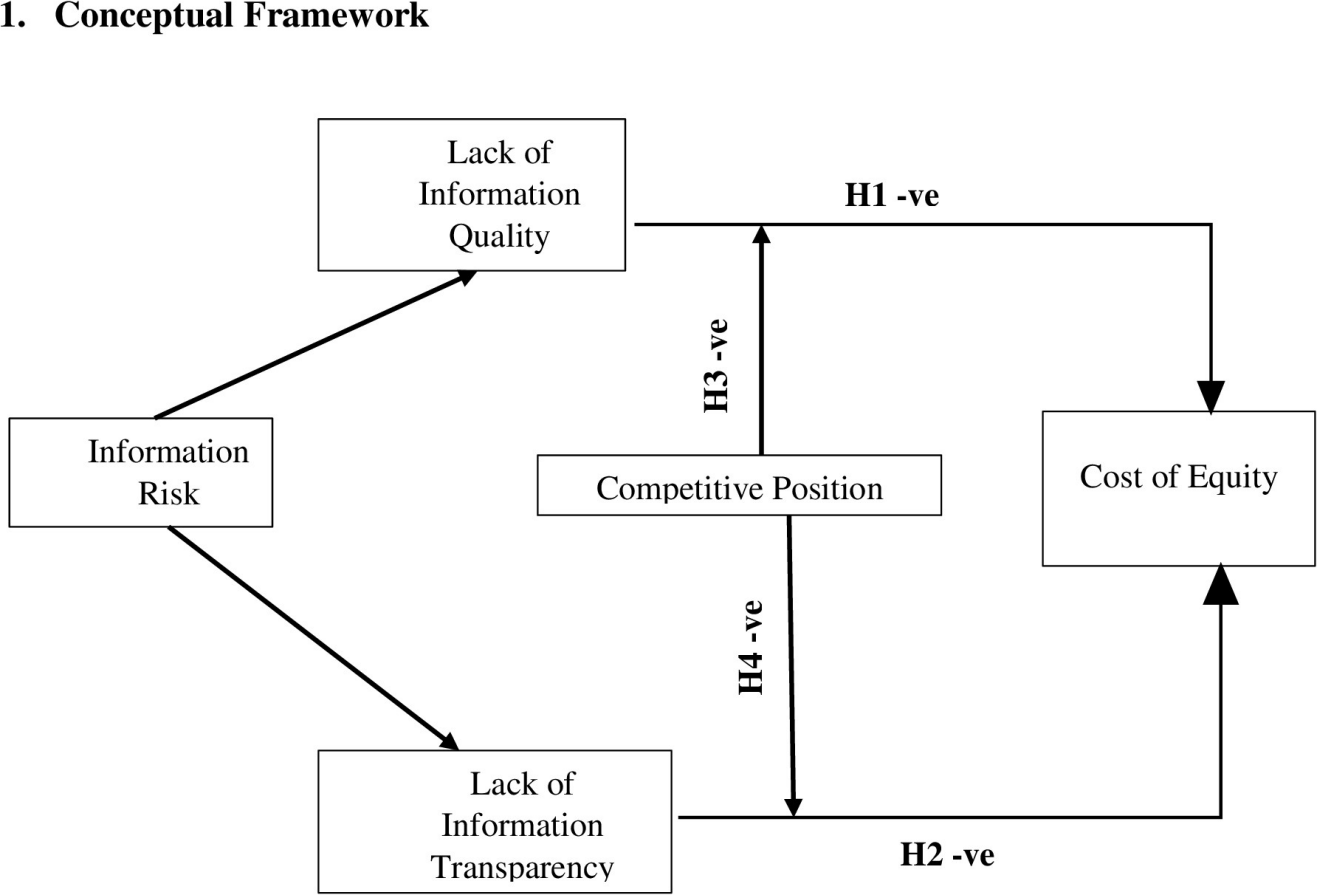
Information risk, competitive position and cost of equity.

However, signaling theory [63], legitimacy theory, and resource dependence theory [64] all contend that companies in dominant market positions may wish to increase their stakes by projecting the image of trustworthy organizations in order to maintain access to essential resources”. [65] argued that having a higher competitive status gives businesses more room to deal with the negative effects of intense market rivalry and a better capability to adapt to changing economic conditions. [65,66] discovered that product market competition lowers company’s cost of capital. Competition, on the other hand, improves not only the efficiency of information disclosure, but also the ability of investors to view and comprehend it. This increases information transparency while lowering the COE. However, a robust theoretical foundation has been provided by the synthesis of these theories. For example, Agency theory and RDT explain how information asymmetry raises perceived risks and COE. Signaling hypothesis and legitimacy theory elaborates how competitive position acts as a signal of stability, trust and reduces COE. This reduced COE improves resource availability and aligns managerial decisions with investment objectives. In the same vein, RDT, signaling Hypothesis and legitimacy theory congregate to illustrate the role of competitive position in amplifying the benefits of condensed information asymmetry, enabling better investment decisions. Based on the preceding theoretical arguments, we propose the conceptual framework as shown in Fig 1.

This conceptual framework illustrating the impact of information risk on cost of equity. Information risk is represented by two distinct forms: lack of information quality and lack of information transparency. The framework proposes that these types of information risk negatively influence the cost of equity, with competitive position acting as a moderating variable. Hypotheses H1 to H4 denote the expected negative relationships among the constructs.

***H1*** *: Lack of information quality increases the Cost of Equity*

***H2*** *: Lack of information transparency increases the Cost of Equity*

***H3*** *: Firm’s competitive position weakens the negative relation between lack of information quality and COE.*

***H4*** *: Firm’s competitive position weakens the negative relation between the lack of information transparency and COE.*

### 2.1. Information risk, competitive position and corporate investment

Capital providers, unlike managers, do not have accurate information about the projected profit potential of a company’s investment decisions. As a result, the cost of capital rises, limiting the firm’s borrowing capacity. Due to financial constraints, firms miss out several investment opportunities, leading to underinvestment [67,68].

The signaling theory states that a company’s competitive position improves the quality of its reporting [69,70]. New share issuance, on the other hand, puts negative signals to investors by reducing the advantages and integrity of current shareholders [58,71].

Firm’s strong competitive position signals inherent strengths and future profitability, mitigating concerns arising from lack of information quality. This competitive advantage reduces uncertainty and perceived risk, making investors more willing to commit capital despite informational shortcomings. Consequently, the negative impact that lack of information quality typically has on corporate investment is weakened. Thus, a robust competitive position lessens investors’ sensitivity to information gap supporting continued investment [61,72–74]. In other words, when there is a lot of competition, it’s easier for investors to keep track of accounting data from multiple companies.

One of the Pakistan’s leading conglomerates, Engro Corporation periodically provides detailed reports on financial disclosure and SDGs by following international standards, for instance, Global reporting initiatives. This proactive disclosure gives signals of reliability to investors and attracts both domestic and foreign investment by reducing the information asymmetry. Consequently, Engro often gets capitals at competitive rates by illustrating the real-world relevance of legitimate theory and signaling theory. This strong competitive standing and transparency of Engro have allowed it to embark on substantial investments in agriculture, energy and chemical sectors to advance sustainable growth.

**Fig 2:**
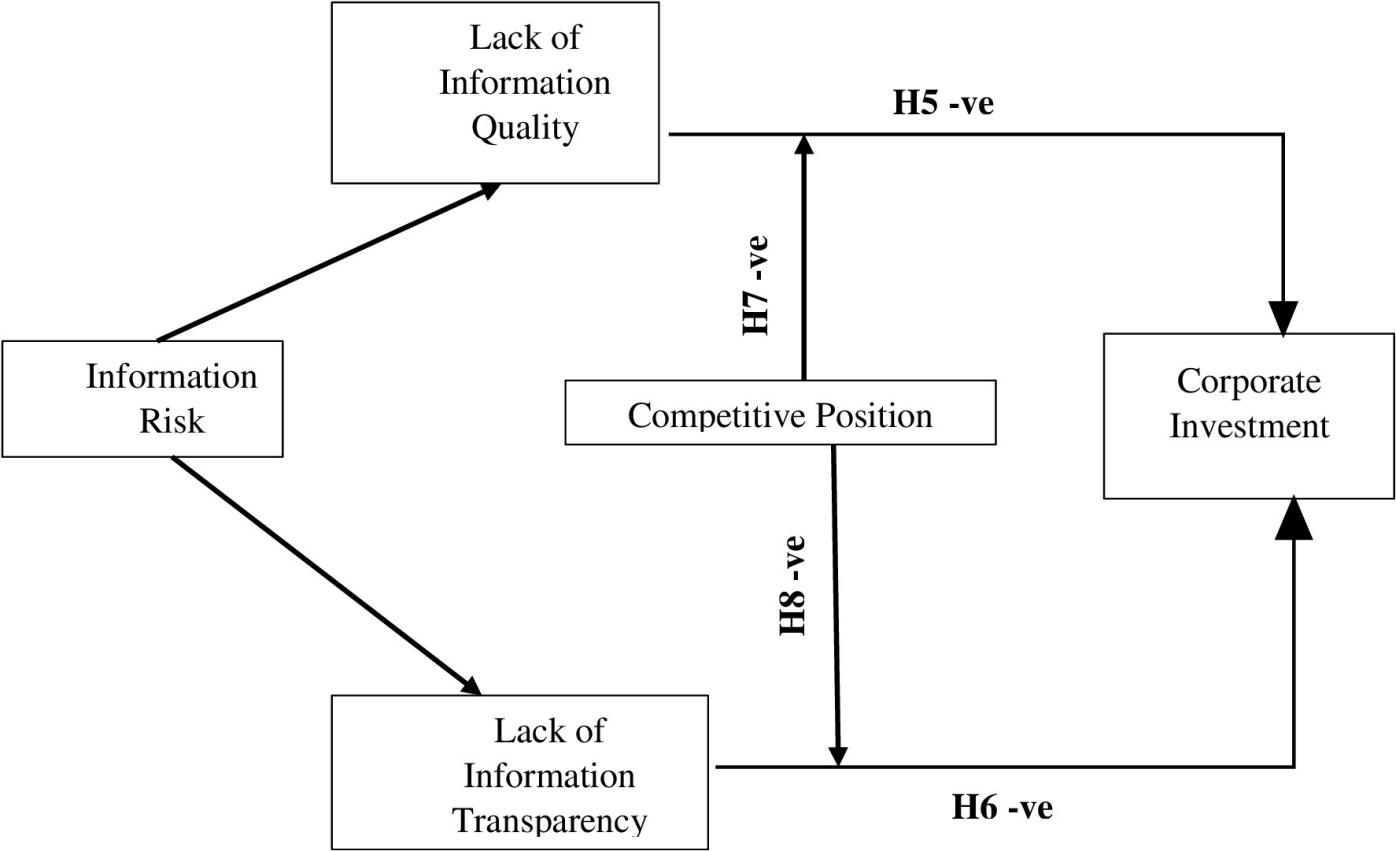
Information risk, competitive position and corporate investment.

In contrast, firms listed on PSX have faced several challenges related to transparency due to inconsistent reporting standards. Firms with weak governance and restricted disclosure practices, for instance, small and mid-sized enterprises experience higher COE due to perceived risk. RDT and legitimacy theory explained how firms can overcome these challenges through strategic transparency, by improving competitive standing and with alignment of societal norms, lower COE and foster investment opportunities. Based on the theoretical foundation, we propose the conceptual framework as shown in Fig 2.

This framework explores how information risk—defined by information quality and transparency—negatively impacts corporate investment, with competitive position serving as a moderator. Hypotheses H5 to H8 outline the expected negative relationships among these variables.

***H5*** *: Lack of information quality decreases the corporate Investment.*

***H6*** *: Lack of information transparency decreases the corporate Investment.*

***H7*** *: Firm’s competitive position weakens the negative relation between lack of information quality and corporate Investment.*

***H8*** *: Firm’s competitive position weakens the negative relation between lack of information transparency and corporate Investment.*

## 3. Methodology

### 3.1. Population and sample

The population of the study consists of companies listed on Pakistan Stock Exchange [PSX]. There are 559 companies listed. Financial firms are excluded. The reason of exclusion of financial firms is based on the highly leveraged capital structure and steering by the regulatory capital requirement. Therefore, the non-financial firms are included in this research for empirical analysis and there are 430 non-financial firms listed on the PSX. The data came from publicly available financial records that are accessed on the respective firms’ official websites. A two-step system GMM dynamic panel estimator technique is utilized to look into the relationship between the concerned variables.

### 3.2. Variable measurement

a ***Information Quality***

The Accrual quality [AQ] is used as a measure of information quality in this study; it was originally developed by [75–77] as follows:


$$\begin{array}{c} TCA_{it}=\beta_{0}+\beta_{1}CFO_{i\left[t-1\right]}+\beta_{2}CFO_{it}+\beta_{3}CFO_{i\left[t+1\right]}+\varepsilon_{it} \end{array}$$ (1)

[78] refined this model by introducing two more accrual factors:


$$\begin{array}{c} TCA_{it}=\beta_{0}+\beta_{1}CFO_{i\left[t-1\right]}+\beta_{2}CFO_{it}+\beta_{3}CFO_{i\left[t+1\right]}\beta_{4}\Delta REV_{it}+\beta_{5}PPE_{it}+\varepsilon_{it} \end{array}$$ (2)

Equation (4) has been predicted for each industrial group in each year. The standard deviation of the residual [captured by equation (2)] over the previous five years determines a firm’s reporting quality, and a high AQ value indicates less quality information available to investors.

b ***Information Transparency***

This study operationalizes information transparency by constructing a measure that depends on the explanatory power of the relationship between returns and earnings, i.e., the degree to which earnings and change in earnings covariate simultaneously with returns on the stock [7,73]. This study measures TRANS by using the two-step estimation process. “This metric for each firm-year is calculated by taking the sum of explanatory power from this regression of return-earnings relationship in two steps”. Regression is carried out in the first phase in accordance with equation (3) as follows:


$$\begin{array}{c} RE{𝐓}_{i,t,t}=a_{0}^{I}+a_{1}^{I}E_{i,j,t}/P_{i,j,t-1}+a_{2}^{I}\Delta E_{i,j,t}/P_{i,j,t-1}+\varepsilon_{i,j,t} \end{array}$$ (3)

“Where RET represents the firm’s yearly return, Et/P t-1 represents earnings divided by the starting year’s price, and E represents the change in earnings.”

“In the subsequent phase, firms are sorted into four portfolios, from smallest to largest on the basis of residual obtained from equation (3). Following that, regression is performed in each portfolio, as displayed in equation (4). Similarly, the adjusted R square in this step reveals how much the earnings and change in earnings co-vary with the returns on each company’s portfolio, as denoted by TRANSIN.”


$$\begin{array}{c} RET_{i,p,t}=a_{0}^{IN}+a_{1}^{IN}E_{i,p,t}/P_{i,p,t-1}+a_{2}^{IN}\Delta E_{i,p,t}/P_{i,p,t-1}+\varepsilon_{i,p,t} \end{array}$$ (4)

“By adding the adjusted R squares from equations (3) and (4), the earning transparency is finally determined”.


$$\begin{array}{c} TRANS_{i,t}=TRANSI_{j,t}+TRANSIN_{p,t} \end{array}$$ (5)

c ***Measurement of Expected Cost of Equity***

The COE can be defined as the required rate of return by equity providers to jeopardize their capital in business. To measure the COE, this study follows the Leary and Roberts [2014], by extending the model with book to market, size and momentum factors given by [79,80], as follows:


$${𝐑}_{ijt}=\;\alpha_{ijt}+{\beta^{𝐌}}_{ijt}*MK{𝐓}_{𝐭}+{\beta^{SMB}}_{ijt}*SM{𝐁}_{𝐭}+{\beta^{HML}}_{ijt}*HM{𝐋}_{𝐭}+{\beta^{MOM}}_{ijt}*MO{𝐌}_{𝐭}+{\beta^{IND}}_{ijt}[\bar{𝐑}{-}_{ijt}-𝐑{𝐅}_{𝐭]}+{\text{ŋ}}_{ijt}$$ (6)

where R_ijt_ refers to the total return on the stock of the firm i in industry j during the month t, MKT_t_ is the excess return on the market, SMB_t_ is the size factor, HML_t_ is the book-to-market factor, MOM_t_ is the momentum factor, and [R_−ijt_ − RF_t_] is the excess return on the equally weighted industry portfolio, minus firm i’s return. The study includes this last factor in the model to confiscate any common stock returns variation across the industry.”

Using historical monthly returns, the study estimates equation (6) on a rolling annual basis for each company. For this, at least 24 months of historical data are needed. For computing the expected COE, the study first estimates equation (6) using the monthly returns from July 2006 to June 2008. Then uses the proposed coefficients obtained by equation (6) and the monthly factor returns from July 2007 to June 2008, the study determines the expected COE of a particular firm for a specific year by estimating the equation (6.1) as follows:


$$\begin{array}{c} R_{ijt}=\;\alpha_{ijt}+{\beta^{M}}_{ijt}*MKT_{t}+{\beta^{SMB}}_{ijt}*SMB_{t}+{\beta^{HML}}_{ijt}*HML_{t}+{\beta^{MOM}}_{ijt}*MOM_{t}+{\beta^{IND}}_{ijt}[\bar{𝐑}{-}_{ijt}-RF_{t}] \end{array}$$ (6.1)

d ***Corporate Investment***

Capital expenditures are used to measure corporate investment [81,82].

e ***Competitive Position***

A firm’s competitive position can be described as the strength of firm relative to industry [83–85]. This study takes the cumulative growth or decrease in market share over a two-year period as a metric to determine the firms’ competitive position [70]. Thus, measure can be operationalized in following way:


$$\begin{array}{c} Competitive\;Position=\;\sqrt{\frac{Market\;Share_{i,t+2}}{Market\;Share_{i,t}}-1}\; \end{array}$$ (7)

where, the negative value means a decrease in competitive position. The market share of the business in the, ’t’ year with the ‘n’ companies in the industry has been calculated as $\frac{Sales_{i,t}}{\sum_{i=1}^{n}Sales_{i,t}}$. Another metric of competitive position such as industry-adjusted ROA is used to ensure robustness of results. This industry-adjusted ROA is determined by subtracting the average ROA from the company’s ROA for the industry [86].

f ***Control Variables***

The natural logarithm of total assets is used to determine size of the firm [87]. Larger firms generally exhibit lower COE due to enhanced operational stability, diverse operational streams and better access to capital market with favorable conditions. Moreover, Investor perceive larger firms as more transparent and stable which reduce information risk [88,89]. By following the [90], this paper uses the market to book ratio in order to control for the differences in growth opportunities. Investors often demand high return from growth oriented firms because of uncertain future cash flows. CF calculated as “net income before extraordinary items plus depreciation, amortization, and research and development [R&D] expenses, scaled by the beginning of year book value of assets”. Firms with robust cash flows are perceived as financially sound, reducing information asymmetry and COE [91]. Beta, gauges firms sensitivity to changes in market and reflects its exposure to systematic risk, key measure of COE in the CAPM. High beta leads to increase in COE and investor will demand high returns to compensate for the additional risk [19,66].

### 3.3. Data estimation method

With regards to frontier markets like Pakistan, data often have significant variability and data gaps which may undermine the performance of conventional methods like difference GMM and fixed Effects. To resolve methodological concerns with panel data, the study uses the system GMM. [92] Proposed the difference GMM, which converts the regressors using the first difference to eliminate unobserved firm-specific fixed effects and does not change over time. In linear dynamic panel data models, the difference GMM produces skewed and poor precision. Because of two factors, it becomes less informative [92]. First, the variables resemble a random walk [92], and second, the unobserved fixed effect grows.

Moreover, the link between information risk and COE is innately dynamic which means that past COE or corporate investment may influence the current information risk. Fixed effects are not suitable for handling dynamic panels with lagged dependent variables. It produces biased and inconsistent estimates in the situation because of the existence of autocorrelation caused by unobserved heterogeneity and natural correlation of error term with lagged dependent value. The potential endogeneity of the dependent variable is controlled by including the lag value of the same variable as the instrument [93]. To address the issue of endogeneity, the analysis used the two-step GMM Dynamic Panel Estimator method as described by [61,94] to ensure the consistency in estimates by addressing the dynamic nature of model, unobserved heterogeneity and mitigate the weak instruments problem. The Hansen J-test ensures that instruments are valid, not overly identified and uncorrelated with error term. Similarly AR [1] and AR [2] further strengthening the robustness of estimation by validating the absence of serial correlation.

### 3.4. Empirical model

#### 3.4.1. Information risk and cost of equity: Role of competitive position.

In order to define the role of competitive position between the relationship of information quality and COE, model equation (8) is analyzed. This moderating role of firm competitive position is analyzed by using two measures of Competitive position; market share and industry adjusted ROA. Therefore, the role of competitive position in concerned variables is first determined by using the Market share as proxy of Competitive position as follow:


$$\begin{array}{c} \begin{array}{cc} COE_{i,t} & =\beta_{o}+\beta_{1}COE_{i,t-1}+\beta_{2}AQ_{i,t-1}+\beta_{3}AQ_{i,t-1}*Marketshare_{i,t-1}+\beta_{4}Marketshare_{i,t-1}\\ & \;+\beta_{5}Size_{i,t-1}+\beta_{6}\beta eta_{i,t-1}+\varepsilon \end{array} \end{array}$$ (8)

“Where dependent variable, COE is the cost of equity of firm i over the period of time, AQ is measure of information quality of firm i at time t, AQ_i, t-1,r_*Market share is the interaction term, Market share is a measure of competitive position of firm i at time t, and rests are the control variables. To avoid the issue of endogeneity, model is made dynamic where lagged value of dependent variable is used as an independent variable.”

Firms are segregated into two parts based on the median value of Market share. Firms which fall below the median value of Market share are considered as low competitive firms, and in case the value is above the median, the firm is categorized as high competitive firm. After dividing the firms into high and low competitive firms, equation (8.1) is applied in both groups.


$$\begin{array}{c} COE_{i,t}=\beta_{o}+\beta_{1}COE_{i,t-1}+\beta_{2}AQ_{i,t-1}+\beta_{3}Marketshare_{i,t-1}+\beta_{4}Size_{i,t-1}+\beta_{5}\beta eta_{i,t-1}+\varepsilon \end{array}$$ (8.1)

This process is repeated by using Industry adjusted ROA to ensure the robustness of segmentation for high and low competitive firms. This robustness is further checked by interaction effect and subsample analysis. Results of this analysis in Tables 3 and 4 confirmed that the moderating effect of competitive position stayed meaningful across different segmentation methods.

**Table 1:** Descriptive statistics.

Variables	Mean	Std. Dev. [SD]	Minimum	Maximum
**Transparency**	0.2419	0.3816	0.1064	1.7085
**Quality**	0.2385	0.3313	0.0015	0.6842
**Market to book ratio**	1.489	2.2475	-9.5687	12.7314
**Beta**	0.4151	0.8115	-1.4301	1.4033
**CF**	0.0775	0.1374	-0.3071	0.5763
**Size**	17.5267	3.1618	1.781	25.2733
**Market Share [Sale]**	0.02397	0.27072	-0.7573	0.6557
**Market Share [ROA]**	-0.01561	0.08910	-0.3545	0.2585
**Cost of Equity**	0.2374	0.8298	0.0571	0. 5053
**Capital Expenditure**	0.0665	0.1076	0.0020	0.1914

**Table 2:** Correlation analysis.

	COE	Investment	Quality	Trans	Market share	Industry Adjust ROA	MB	Size	Beta	CF
**COE**	1.000									
**Investment**	0.036	1.000								
**Quality**	0.091	-0.092	1.000							
**Trans**	-0.086	0.046	-0.049	1.000						
**Competition**	-0.167	0.054	-0.327	0.036	1.000					
**Industry Adjust** **ROA**	-0.038	0.22	-0.068	0.011	0.752	1.000				
**MB Ratio**	-0.139	0.123	0.088	0.037	0.053	0.107	1.000			
**Size**	-0.032	0.079	-0.065	0.108	-0.029	-0.079	-0.138	1.000		
**Beta**	0.063	-0.046	0.032	-0.016	0.098	0.143	-0.043	-0.04	1.00	
**CF**	-0.166	0.134	0.158	0.102	0.285	0.022	0.311	-0.107	0.028	1.000

**Table 3:** Information risk, competitive position and COE.

Variable	Panel A: Quality			Panel B: Transparency		
	COE	High Market share	Low Market share	COE	High Market share	Low Market share
**Constant**	1.4176** [0.6045]	1.7867** [0.2596]	0.9589*** [0.2628]	0.7271 [0.5242]	8.7270*** [2.2644]	0.4948 [0.3983]
**COE**_**t-1**_	-0.4543*** [0.1018]	-0.7523*** [0.0214]	0.0078** [0.0355]	0.2915** [0.2414]	-0.4940** [0.1795]	-0.0955** [0.0460]
**Quality**	0.0031** [0.0015]	-0.0626 [0.0692]	0.0097** [0.0035]			
Quality* **Market share**	-0.084*** [0.0036]					
**Transparency**				-0.0659** [0.3404]	0.3815** [0.1300]	0.3006* [0.2179]
Trans* **Market share**				-0.1764*** [0.2097]		
**Market share**	-0.1211*** [0.0298]	0.0736*** [0.0076]	0.4553* [0.2449]	-0.0021* [0.1583]	0.0390* [0.0227]	0.0238** [0.2002]
**Beta**	0.2409*** [0.0496]	0.3563*** [0.0066]	0.0337 [0.0253]	-0.0867 [0.1529]	-0.2553** [0.0959]	-0.0502** [0.0230]
**Size**	-0.0516 [0.0339]	-0.0734*** [0.0171]	-0.0310* [0.0177]	-0.0197 [0.0272]	-0.4848*** [0.1354]	-0.0173** [0.0214]
**AR 1**	0.004	0.024	0.055	0.007	0.002	0.012
**AR 2**	0.203	0.169	0.272	0.351	0.668	0.253
**Sargan/Hansen**	1.040/ 0.395	0.241/ 0.599	0.388/ 0.305	0.238/0.225	0.122/ 0.392	0.639/ 0.675
**No. of Instruments**	120	186	192	118	184	186
**No. of Groups**	370	370	370	370	370	370

**Table 4:** Information risk, competitive position and COE.

Variable	Panel A: Quality			Panel B: Transparency		
	COE	High ROA	Low ROA	COE	High ROA	Low ROA
**Constant**	3.7476**	1.0578** [0.4806]	-0.8856** [0.4090]	-0.6973 [0.9437]	-0.4115 [1.1595]	-0.8193 [0.6209]
**COE**_**t-1**_	-0.4461*** [0.1049]	0.1302*** [0.0191]	-0.2238** [0.0714]	0.5674** [0.2712]	0.2171 ** [0.1076]	-0.2548* [0.1386]
**Quality**	0.0151** [0.0060]	-0.0213** [0.0006]	0.0275* [0.0869]			
Quality*Ind **Adj ROA**	-0.0711*** [0.0271]					
**Transparency**				-0.5910* [0.3134]	-0.2196 ** [0.1058]	-0.1643** [0.0823]
Trans* Ind Adj ROA				-0.0348* [5.5165]		
**Ind Adj ROA**	0.4580 [0.6448]	-0.4828* [0.2470]	0.6661*** [0.3918]	-0.6617* [1.5764]	-0.8073* [0.4307]	0.5746*** [0.7172]
**Beta**	0.1579* [0.0643]	0.1262** [0.0453]	0.1224*** [0.0289]	-0.1347 [5.5165]	0.1765** [0.0585]	0.0627 [0.1061]
**Size**	-0.1980** [0.0648]	-0.0512* [0.0283]	0.0897*** [0.0240]	0.0313 [0.0452]	0.0374 [0.0683]	0.0709** [0.0354]
**AR 1**	0.008	0.027	0.030	0.003	0.008	0.002
**AR 2**	0.131	0.405	0.741	0.125	0.197	0.493
**Sargan/Hansen**	0.105/0.219	0.745/0.603	0.415/0.442	0.304/ 0.174	0.863/ 0.657	0.505/ 0.691
**No. of Instruments**	122	83	83	121	82	81
**No. of Groups**	370	370	370	370	370	370

#### 3.4.2. Information risk, competitive position [Market share] and corporate investment.

The effect of firms’ competitive position on the relationship between less information quality and corporate investment is determined as follow:


$$\begin{array}{c} I_{i,t}={\mathrm{\backslash boldmath}\beta }_{o}+\beta_{1}I_{i,t-1}+\beta_{2}Q_{i,t-1}+\beta_{3}AQ_{i,t-1}*Q_{i,t-1}+\beta_{4}AQ_{i,t-1}+\beta_{5}Size_{i,t-1}+\beta_{6}\beta eta_{i,t-1}+\beta_{7}CF+\varepsilon \end{array}$$ (8.2)

“Where, the dependent variable, I_i, t,_ is firm i’s investment in year t; CAPX _i,t_ is used as measure of corporate investment. The independent variables are: Qi, _t−1_ is Tobin’s Q and AQ_i, t−1_ is a measure of information quality. The response of investment-Q sensitivity to accrual quality is characterized by the coefficient value of interaction term, AQ × Q”. The set of control variables [CONTROLS] includes the cash flow [CFi, t], *Size* is the natural logarithm of’ total assets of firm i at time, Beta as a measure of systematic risk. This model further explores whether the relationship between Investment-Q sensitivity and opaque information is also influenced by firm’s competitive position. For this reason, this study interacted the INFO × Q with firm’s competitive position by using two proxies: Market Share and industry adjusted ROA. The parameter of our interest is value of interaction term Q*AQ* Market share which show the effect of competitive position between the relationship of Investment-Q sensitivity and information quality as follow:


$$\begin{array}{c} I_{i,t}=\beta_{o}+\beta_{1}I_{i,t-1}+\beta_{3}Q_{i,t-1}+\beta_{4}AQ_{i,t-1}+\beta_{5}Q_{i,t-1}*A*Marketshare_{i,t-1}+\beta_{6}Size_{i,t-1}+\beta_{7}\beta eta_{i,t-1}+\beta_{8}CF+\varepsilon \end{array}$$ (8.3)

#### 3.4.3. Information risk [Information transparency], competitive position [Market Share] and COE.

The moderating role of competitive position, using market share as proxy, between information transparency and COE is analyzed by estimating the following model:


$$\begin{array}{c} \begin{array}{cc} COE_{i,t} & =\beta_{o}+\beta_{1}COE_{i,t-1}+\beta_{2}Trans_{i,t-1}+\beta_{3}Trans_{i,t-1}*Marketshare_{i,t-1}\\ & \;+\beta_{4}Marketshare_{i,t-1}+\beta_{5}Size_{i,t-1}+\beta_{6}\beta eta_{i,t-1}+\varepsilon \; \end{array} \end{array}$$ (8.4)

“Where *Market share is the interaction term, Market share is a measure of competitive position of firm i at time t, and rests are the control variables. To avoid the issue of endogeneity, model was made dynamic where lagged value of dependent variable is used as an independent variable”.

Moreover, firms are segregated into two parts based on the median value of Market share and then, equation (8.4) is applied in both groups.


$$\begin{array}{c} COE_{i,t}=\beta_{o}+\beta_{1}COE_{i,t-1}+\beta_{2}Trans_{i,t-1}+\beta_{3}Trans_{i,t-1}+\beta_{4}Size_{i,t-1}+\beta_{5}\beta eta_{i,t-1}+\varepsilon \end{array}$$ (8.5)

#### 3.4.4. Information risk, competitive position [Market share] and corporate investment.

Further, the effect of information transparency on corporate investment is investigated by using the following model:


$$\begin{array}{c} I_{i,t}=\beta_{o}+\beta_{1}I_{i,t-1}+\beta_{2}Q_{i,t-1}+\beta_{3}Trans_{i,t-1}+\beta_{4}Trans*Q_{i,t-1}+\beta_{5}Size_{i,t-1}+\beta_{6}\beta eta_{i,t-1}+\beta_{7}CF+\varepsilon \end{array}$$ (8.6)

This model examines that whether the impact of information transparency on Investment-Q sensitivity is also affected by firm’s competitive position. For this purpose, we respectively interact, INFO × Q. The parameter of our interest is value of interaction term Q*Trans* Market share which show the effect of competitive position on the relation between information transparency and Investment-Q sensitivity.


$$\begin{array}{c} \begin{array}{cc} I_{i,t} & =\beta_{o}+\beta_{1}I_{i,t-1}+\beta_{3}Q_{i,t-1}+\beta_{4}Trans_{i,t-1}+\beta_{5}Q_{i,t-1}*Trans*Marketshare_{i,t-1+}\\ & \;+\beta_{6}Marketshare_{i,t-1+}+\beta_{7}Size_{i,t-1}+\beta_{8}\beta eta_{i,t-1}+\beta_{9}CF+\varepsilon \end{array} \end{array}$$ (8.7)

## 4. Results and discussions

### 4.1. Descriptive statistics

Table 1 presents the summary statistics for the variables utilized in this study spanning from 2007 to 2022. Generally, transparency levels hover around 24 percent, while information quality stands at 23 percent. The notable disparity between the lowest and highest values for information quality and transparency underscores the broad spectrum of disclosure and transparency practices among the firms listed on the PSX. The market-to-book ratio, indicating growth opportunities, has a mean value of 1.48, whereas the mean book-to-market value is 0.7649. Beta, serving as a measure of systematic risk, indicates a mean value of 0.4151 with a standard deviation of 0.8115. Long-term debt, in terms of leverage, comprises an average of 17% of a company’s assets according to the mean value. The cash flow ratio exhibits a mean value of 0.0775 with a standard deviation of 0.1374. The average firm size is 17.526 with a standard deviation of 3.161%. Furthermore, the mean value for the firm’s competitive position is 0.023 with a standard deviation of 0.2707. The industry-adjusted return on assets [ROA] has an average value of -0.0156 with a standard deviation of 0.2585. On average, the cost of equity [COE] is around 23% with a standard deviation of 0.8298. The range between the minimum and maximum values illustrates a significant disparity in COE among the firms listed on the PSX. The average firm investment stands at 0.0665 with a standard deviation of 0.1076.

### 4.2. Correlation matrix

The correlation among the variables is reported in Table 2 which shows that problem of multi-collinearity does not exist because the explanatory variables are independent in nature.

### 4.3. Information risk, competitive position and COE

Table 3 depicts the effect of lack of information quality and transparency on COE while accounting for the moderating effect of firms’ competitive position. In first column of Panel A, this link is established by using the entire sample, where the coefficient value of the interaction indicates a negative relationship with COE. These results confirm our hypothesis and support the findings of [44,73,95,96], who suggest that firms demonstrate the strength of their risk management by being more transparent, and they strive to maintain access to financial resources. This level of transparency is consistent with uncertainty reduction theory [84]. Furthermore, firms with a declining market share may be reluctant to disclose concerns because doing so could result in higher political and equity costs [63,81].

These results are also confirmed by third and fourth columns of Table 3, where firms are segregated into two groups based on their competitive position measured by market share. Table 3 indicates that in highly competitive firms, the coefficient value of accrual quality is negative and significant, whereas it is positive in less competitive firms. The results indicate that firms in highly competitive industry face lower COE because the firms that do well in the market demonstrate their resilience through enhanced transparency to preserve their reputation and stakeholders’ trust [44]. In addition, BOD will preclude the managers from acting opportunistically with better supervision and the issue of high compensation may be addressed by reducing the information asymmetry [97]. Similarly, when there is positive correlation between insider and outside information, it enhances the market liquidity, market efficiency and trading volume which ultimately reduce the COE [98].

Further, in Panel B, the association between lack of information transparency and COE is determined. In first column of Panel B, this relationship is first established by examining the entire sample where the coefficient interaction term with competitive position shows that greater information transparency and strong market competition decreases the firms’ COE. Reason is strong competitive position required improving the standards and reliability of accounting information [93,99–101]. These results are consistent with legitimacy theory, signaling theory and resource-based theory.

Separating the entire sample into high and low competitive firms, however, also confirmed this relationship, with the results shown in the 6th and 7th columns of Table 3. The results indicate that highly competitive firms experience less COE because of the competition, when an investor gains information from various sources outside the business and gets a deep understanding of the company by comparing different firms; it increases the capacity of the investors to understand financial information [57].

### 4.4. Information risk, competitive position and COE [Sale]

Table 4 exhibit the effect of lack of information quality and information transparency on COE by considering the moderating role of firm competitive position. Here, the industry adjusted ROA is used as another measure of competitive position. The results obtained by second proxy of competitive position are similar to the first proxy who confirms the moderating role of firm competitive position between the information risk and COE.

### 4.5. Information risk, competitive position and corporate investment

Table 5 exhibits the results related to effect of information risk on corporate investment. First, Panel A establishes the link between corporate investment and opaque information. Second, the role of competitive position in the relationship under consideration is examined.

**Table 5:** Information risk, competitive position and corporate investment.

Variables	Panel A: Information Quality			Panel B: Information Transparency		
	Quality			Transparency		
		ROA	Market share	Investment	ROA	Market share
**Constant**	-9.4079*** [1.3775]	-0.1559 [0.1085]	-0.6625*** [0.1634]	-7.0304*** [0.2394]	0.5505*** [0.1501]	-7.8646*** [0.3196]
**Expenditures** **t-1**	0.0208** [0.0085]	0.0038*** [0.0022]	0.0101*** [0.0007]	0.0153*** [0.0012]	0.0004*** [0.0008]	0.0206*** [0.0014]
**Q**	0.2082** [0.0870]	0.0115*** [0.0092]	0.0209*** [0.0105]	0.0696*** [0.0118]	0.1074*** [0.0063]	0.0872*** [0.0121]
**Quality**	-0.1664*** [0.0497]	-0.0026 [0.0009]	0.0024*** [0.0004]			
Quality*Q	-0.5858*** [0.1853]					
**Trans**					0.2054** [0.0900]	0.4998*** [0.0552]
Trans* Q				0.4890*** [0.0510]		
Quality* Q* **Ind Adj ROA**		0.1529** [0.0448]				
**ROA**		0.3329** [0.1361]			0.2621 [0.2123]	
Trans*Q* **Ind Adj ROA**					0.6655** [0.2856]	
**Sales**			0.0838** [0.0160]			0.0401*** [0.0095]
Quality*Q* **Sales**			0.0063*** [0.0004]			
Trans*Q* **Sale**						0.5752*** [0.0110]
**CF**	0.0952*** [0.0117]	0.0467*** [0.0045]	0.0990*** [0.0044]	0.2769*** [0.0032]	0.0100** [0.0048]	0.2768*** [0.0040]
**AR 1**	0.742	0.007	0.009	0.009	0.005	0.006
**AR 2**	0.934	0.204	0.262	0.234	0.481	0.220
**Sargen/Hansen**	1.000/ 0.817	1.000/0.836	1.000/0.171	0.128/0.114	1.000/0.999	0.223/0.241
**No. of Groups**	124	124	123	123	123	122
**No. of Instruments**	370	370	370	370	370	370

Results in column 1 show that investment is positively associated with Tobin’s Q, and investment Q sensitivity is negatively correlated with opaque information. This opaque information also has negative association with corporate investment. This is due to the fact that low information quality aggravates information asymmetry between managers and investors which may result in investment inefficiency.

Moreover, the effect of firm competitive position on the relationship between opaque information and corporate investment is analyzed. In 3^rd^ column of Table 5, where the industry adjusted ROA is used as an alternative measure of firm’s competitive position, the coefficient value of interaction term [Quality * ROA] is statistically significant [β = 0.1529, p < 0.05]. This coefficient value suggests that firm’s competitive position is negatively allied with opaque information in order to increase the investment. The rationale behind this finding is that quality of disclosure is higher because the degree of competition influences voluntary disclosure and limits the manager’s ability to exploit earnings as well [102]. As a result, companies are motivated to grow their market share by constantly developing new products and investing resources in a highly competitive industry.

Similarly, in 3^rd^ column of Table 5 the effect of competitive position is analyzed by using the second proxy, market share. The coefficient value of interaction term Quality*Q*Market share [β = 0.0063, p < 1%] also shows positive association with investment. The coefficient value of market share is smaller but positive as compared to the coefficient value of adjusted ROA.

Furthermore, the effect of information transparency on corporate investment is determined by considering the moderating role of investor attention. The coefficient value of transparency [β = 0.2054, p < 5%] show the positive and significant relation with corporate investment. Moreover, when the coefficient value of transparency interacts with ROA as denoted by Trans* Q*ROA, the increased coefficient value of this interaction term [β = 0.6655, p < 5%] indicates that a firm with strong competitive position increases disclosure, as disclosure in a competitive market may be used as a weapon to win over the credibility and to make certain the continuing access to financial means [64].

Likewise, the increased coefficient value of trans*Q*sale [β = 0.5752, p < 1%] as compared to the coefficient value of Trans [β = 0.4998, p < 1%] demonstrate that companies with rising market share are committed to greater transparency to improve operational sustainability by ensuring ongoing access to key resources. Such increased accountability conveys a signal to stakeholders that the company has preserved its ethical principles while gaining a competitive position [44].

In recap, the reason of differences between the relationship among information risk, COE and corporate investments stem from variation in access to capital, market confidence and transparency. Adverse effects of information risk are mitigated by high competitive firms leveraging strong signaling, operational efficiency and heterogeneous funding sources. Taking the example of Engro Corporation in Pakistan publishes annual reports on governance and SDGs to augment transparency, plummeting information risk and COE. On the other hand, low competitive firms are susceptible to resource constraints and weak market positioning. These initiatives accentuate the pivotal role of competitive standing in revamping financial and investment returns, specifically in emerging markets.

### 4.6. Robustness check

In order to check the moderating role of competitive positioning, different robustness analyses are performed. First, firms are segregated into high and low competitive firms based on the two indicators, e.g., Market share and Industry adjusted ROA. Median split is used as threshold which categorized the firm as “high competitive position” fall above the median value and those below as “low competitive position”. Moreover, interaction effects, subsample analysis, exclusion of outliers and endogeneity checks are applied to robustness of findings. Sub-sample analysis is carried out by performing separate regressions for the high and low firms to corroborate that moderating effect of competitive position on information risk and COE are consistent across distinct subsamples. Similarly, critical points in metrics like market share or industry adjusted ROA are excluded to prevent outliers to distort results. In Nutshell, system GMM leveraged lagged variables as a tool for instrumentation to tackle for potential endogeneity in the segregation process and verified that competitive position is exogenous to other variables like COE or investment decisions.

## 5. Conclusion

The purpose of this study is to explore the moderating role of firm competitive position on the relationship between information risk and COE of firms listed on PSX. This study also examines the moderating role of firm’s competitive position on the relationship between information risk and Corporate Investment. For this purpose, data is collected from all the non-financial firms listed on PSX during the period from 2007 to 2022. To deal with potential endogeneity issue, results are estimated using the two-step System GMM. Results show that investors charge the premium for bearing the risk of information disadvantage.

The findings show that on average, the value of accrual quality and transparency is 0.23 and 0.24. This demonstrates that earning information content in Pakistan is very susceptible to opportunistic earning management while the low figure of transparency clearly draws attention towards lack of transparency. Despite growing calls for greater transparency and accountability, we discover evidence of middling disclosures. The findings are consistent with those of Abraham and [103,104], who argued that companies continue to give metaphorical and formulaic disclosures to policy makers and stakeholders year after year as a form of eyewash. Moreover, the main objective of this study is to explore the moderating role of firm competitive position between the less information quality and COE. Results indicate that competitive position improves the information quality and information transparency which reduces the COE.

Findings demonstrate that a firm’s improved competitive position increases not only the quantity but also the clarity of disclosure. When annual reports convey or address positive performance, management is more motivated to make them easy to read. In other words, Competitive status makes the information climate more transparent, and companies that are willing to compete are more generous and transparent in their disclosure to ensure their access to key financial resources by giving the signal of their strength of risk management. In addition, companies enhance their credibility and build investor’s trust in uncertain times by releasing ‘rich’ and more detailed’ disclosures [uncertainty reduction theory]. This increased investor trust decreases the expense of the agency cost and allows firms to access external capital at a lower price that reduces COE and increases investment. Thus, this study showcasing that information risk escalates the COE and hindering corporate investment, while a solid competitive position offsets this effect. The findings are beneficial for emerging markets, such as Pakistan, which implies that attempts to enhance transparency in disclosure policies may prove fruitful not only in terms of governance but also strategic aspects, such as the competitive position of companies, are factored in. Policy makers should bolstering governance practices, fostering stock market growth and alleviating market distortions through enhanced data openness and accessibility. These measures complement the study findings by addressing the problem of information asymmetry, reducing COE while enabling strategic corporate investment to stimulate economic development.

Building on the findings of this study, several avenues for future research are emerging. First, qualitative investigations could complement the quantitative results presented here. Engaging in interviews with executives and decision-makers might illuminate the strategic factors influencing corporate investment decisions and how firms perceive and manage information risk in relation to their competitive stance. Such insights would prove invaluable for devising more effective risk management strategies.

Second, exploring the impact of technological advancements, such as block chain and artificial intelligence, on mitigating information risk and its implications for COE and corporate investment offers fertile ground for future inquiry. As these technologies continue to evolve, their potential to revolutionize business operations and strategic decision-making processes warrants closer scrutiny.

Finally, there exists ample opportunity for future research to delve deeper into the intricate relationships among information risk, competitive positioning, COE, and corporate investment. By addressing these recommendations, forthcoming studies can make significant contributions to academic literature and offer practical guidance for practitioners seeking to optimize their investment strategies amidst information risks.
